# Charting a course for genetic diversity in the UN Decade of Ocean Science

**DOI:** 10.1111/eva.13224

**Published:** 2021-05-04

**Authors:** Alex Innes Thomson, Frederick I. Archer, Melinda A. Coleman, Gonzalo Gajardo, William P. Goodall‐Copestake, Sean Hoban, Linda Laikre, Adam D. Miller, David O’Brien, Sílvia Pérez‐Espona, Gernot Segelbacher, Ester A. Serrão, Kjersti Sjøtun, Michele S. Stanley

**Affiliations:** ^1^ Scottish Association for Marine Science Scottish Marine Institute Oban UK; ^2^ Southwest Fisheries Science Centre NOAA La Jolla CA USA; ^3^ New South Wales Fisheries National Marine Science Centre Coffs Harbour NSW Australia; ^4^ National Marine Science Centre Southern Cross University Coffs Harbour NSW Australia; ^5^ Oceans Institute and School of Biological Sciences University of Western Australia Crawley WA Australia; ^6^ Laboratory of Genetics, Aquaculture & Biodiversity Universidad de Los Lagos Osorno Chile; ^7^ Centre for Tree Science The Morton Arboretum Lisle IL USA; ^8^ The Wildlife Analysis Unit The Swedish Environmental Protection Agency Stockholm Sweden; ^9^ School of Life and Environmental Sciences Centre for Integrative Ecology Deakin University Geelong Vic Australia; ^10^ Deakin Genomics Centre Deakin University Geelong Vic. Australia; ^11^ Scottish Natural Heritage Inverness UK; ^12^ The Royal (Dick) School of Veterinary Studies and The Roslin Institute Midlothian UK; ^13^ Chair of Wildlife Ecology and Management University Freiburg Freiburg Germany; ^14^ CCMAR Centre of Marine Sciences Faculty of Sciences and Technology University of Algarve Faro Portugal; ^15^ Department of Biological Sciences University of Bergen Bergen Norway

**Keywords:** adaptation, biodiversity, ecosystem resilience, genetic diversity, marine, ocean, restoration, UN Decade

## Abstract

The health of the world's oceans is intrinsically linked to the biodiversity of the ecosystems they sustain. The importance of protecting and maintaining ocean biodiversity has been affirmed through the setting of the UN Sustainable Development Goal 14 to conserve and sustainably use the ocean for society's continuing needs. The decade beginning 2021–2030 has additionally been declared as the UN Decade of Ocean Science for Sustainable Development. This program aims to maximize the benefits of ocean science to the management, conservation, and sustainable development of the marine environment by facilitating communication and cooperation at the science–policy interface. A central principle of the program is the conservation of species and ecosystem components of biodiversity. However, a significant omission from the draft version of the Decade of Ocean Science Implementation Plan is the acknowledgment of the importance of monitoring and maintaining genetic biodiversity within species. In this paper, we emphasize the importance of genetic diversity to adaptive capacity, evolutionary potential, community function, and resilience within populations, as well as highlighting some of the major threats to genetic diversity in the marine environment from direct human impacts and the effects of global climate change. We then highlight the significance of ocean genetic diversity to a diverse range of socioeconomic factors in the marine environment, including marine industries, welfare and leisure pursuits, coastal communities, and wider society. Genetic biodiversity in the ocean, and its monitoring and maintenance, is then discussed with respect to its integral role in the successful realization of the 2030 vision for the Decade of Ocean Science. Finally, we suggest how ocean genetic diversity might be better integrated into biodiversity management practices through the continued interaction between environmental managers and scientists, as well as through key leverage points in industry requirements for Blue Capital financing and social responsibility.

## 1 INTRODUCTION

The marine environment covers 71% of the world's surface, and its coastal areas are home to an estimated 44% of the world's population (UN Ocean Conference, 2020). The biodiversity of our oceans is vital to coastal communities and wider society around the world, providing essential food, income, and bio‐products (FAO, 2019b; Jouffray et al., 2020), as well as supporting critical socioeconomic and cultural values (Bennett et al., 2016; Díaz et al., 2018), and influencing global biogeochemical cycles (Henley et al., 2020; Macreadie et al., 2019). The improved management and protection of marine biodiversity have thus been recognized as a priority area for governments and stakeholders and has sparked a wave of international pledges aimed at restoring biodiversity and ecosystem function globally (CBD, 2020a,2020b; IOC, 2019; Stuchtey et al., 2020; UN Global Compact, 2020; UNEP/FAO, 2020).

The UN Decade of Ocean Science for Sustainable Development (hereafter “the Decade”) represents a major international framework established to support the sustainable development of the world's oceans beyond 2021 (Claudet et al., 2020; IOC, 2019; Ryabinin et al., 2019). The framework highlights the importance of the science–policy interface for strengthening the management of ocean ecosystems and services, emphasizing not only the need for improved understanding and transformative action but also the need for fairer and more equitable access and stewardship of the marine environment. A primary focus in the framework is the maintenance of ocean biodiversity to support the long‐term function and resilience of the marine environment, as well as the sustainable development of the ocean for socioeconomic needs.

While the draft version of the Decade Implementation Plan addresses the conservation and restoration of both species and ecosystem components of biodiversity, a significant omission in the draft is the critical need for the protection, monitoring, and maintenance of intraspecific genetic diversity (IOC, 2020). To highlight this gap, an international panel of leading researchers in marine biodiversity and conservation genetics produced a document directed to the Intergovernmental Oceanographic Commission (IOC) emphasizing the significance of this omission, and the importance of including key objectives focused on the preservation of genetic diversity and evolutionary potential in the final version of the Implementation Plan (available in Supplementary Data). Here, we provide a detailed discussion, based around the document written to the IOC, on the critical need to preserve genetic diversity in order to maintain the health and function of marine ecosystems. We follow this up by highlighting the contributions of genetic diversity and its assessment to key areas of marine research, development, and management that underpin the Decade program. Finally, we discuss critical areas for policy development and research–policy collaboration, and identify points of leverage where genetic diversity management can be encouraged and implemented in industry and the private sector.

## 2 ECOLOGICAL AND EVOLUTIONARY ROLES OF GENETIC DIVERSITY

Genetic diversity represents one of the three fundamental pillars of biodiversity, alongside species diversity and ecosystem diversity (Noss, 1990). Genetic diversity provides the basis for adaptation and evolutionary change, and underpins the resilience and functionality of aquatic and terrestrial ecosystems (Hoffmann et al., 2017; Raffard et al., 2019).

At an evolutionary scale, genetic diversity offers the basic units for adaptive changes that enable populations to respond to shifts in their environment (Jump et al., 2009; Schindler et al., 2015). Adaptation is often derived from standing genetic variation in local populations, as well as from the exchange of genetic variants among populations spanning environmental gradients (Bitter et al., 2019; Hermisson & Penning, 2017; Nosil et al., 2019). Maintaining genetic diversity to support adaptability is particularly pertinent given projections of rapid climate change, as well as increasing stresses from environmental pressures such as habitat fragmentation, ecosystem degradation, and the unprecedented spread and proliferation of invasive species (Babcock et al., 2019; Díaz et al., 2019; Hoegh‐Guldberg et al., 2017; Hoffmann & Sgrò, 2011; Norberg et al., 2012; Wilson et al., 2020). Consequently, the conservation and maintenance of genetic diversity should be an essential management priority for ensuring the future resilience and adaptive potential of populations worldwide.

At a community level, functional genetic diversity has an important role in ecosystem productivity, stability, and function, comparable to that of species diversity (Crutsinger et al., 2006; Raffard et al., 2019). Increased intraspecific genetic diversity has been associated with higher productivity, growth, and ecosystem functions, such as nutrient turnover, in numerous systems, including in marine seagrass beds and diatom blooms (Karbstein et al., 2020; Meilhac et al., 2019; Salo & Gustafsson, 2016; Sjöqvist & Kremp, 2016). Higher genetic diversity can also increase community stability and the stability of ecosystem functions such as productivity over time (DuBois et al., 2021; Meilhac et al., 2019; Prieto et al., 2015; Salo & Gustafsson, 2016). The positive effects of genetic diversity on productivity and ecosystem function are often more prominent under stress conditions, where the effects of complementarity between genotypes can facilitate productivity and resilience (Chalmandrier et al., 2017; DuBois et al., 2021; Evans et al., 2017; Reusch et al., 2005). Many of the effects of genetic diversity on community function and resilience can be explained in part by its contribution to functional trait diversity within species and populations, and its role in increasing community stability, resilience, and facilitation under stress (Bongers et al., 2020; Chalmandrier et al., 2017; Karbstein et al., 2020; Wood et al., 2017). Increased genetic diversity has been suggested to have a particularly strong influence on productivity and community function in foundation species and primary producers (Raffard et al., 2019; Reusch & Randall Hughes, 2006; Wernberg et al., 2018) (Box 1). Enhanced productivity and greater niche availability from increased functional variation in primary producers can in turn affect associated macrofaunal abundance, species richness, and β‐diversity in the wider community and over multiple trophic levels (Barantal et al., 2019; Crutsinger et al., 2006; Hahn et al., 2017; Koricheva & Hayes, 2018; Reusch et al., 2005; Reynolds et al., 2012).

BOX 1 **Ecosystem functions of genetic diversity in seagrass habitats—an example of genetic diversity function in the marine environment. Photos: Zostera marina and associated fauna in a seagrass habitat, Oban, Scotland (Alasdair O’Dell)**

**Table:** 

Seagrasses represent important foundation species in coastal ecosystems, contributing to local fisheries, sediment stability, nutrient turnover, and carbon sequestration services in their environment (Nordlund et al., 2016; Salinas et al., 2020). Genetic diversity in seagrass systems has been positively associated with productivity, as well as production and community stability over time and under fluctuating environmental conditions (DuBois et al., 2021; Reusch et al., 2005; Salo & Gustafsson, 2016). In particular, higher genetic diversity in seagrass meadows has been found to increase the density of shoots per plot, in turn driving increases in biomass, productivity, and macrofaunal abundance (Ehlers et al., 2008; Reusch et al., 2005; Reynolds et al., 2012). Genetic diversity has also been shown to increase resilience, recovery, and productivity in seagrass under a range of environmental stressors, including shading, sedimentation, and temperature stress (Evans et al., 2017; Plaisted et al., 2020; Ehlers et al., 2008; Reusch et al., 2005; DuBois et al., 2021).	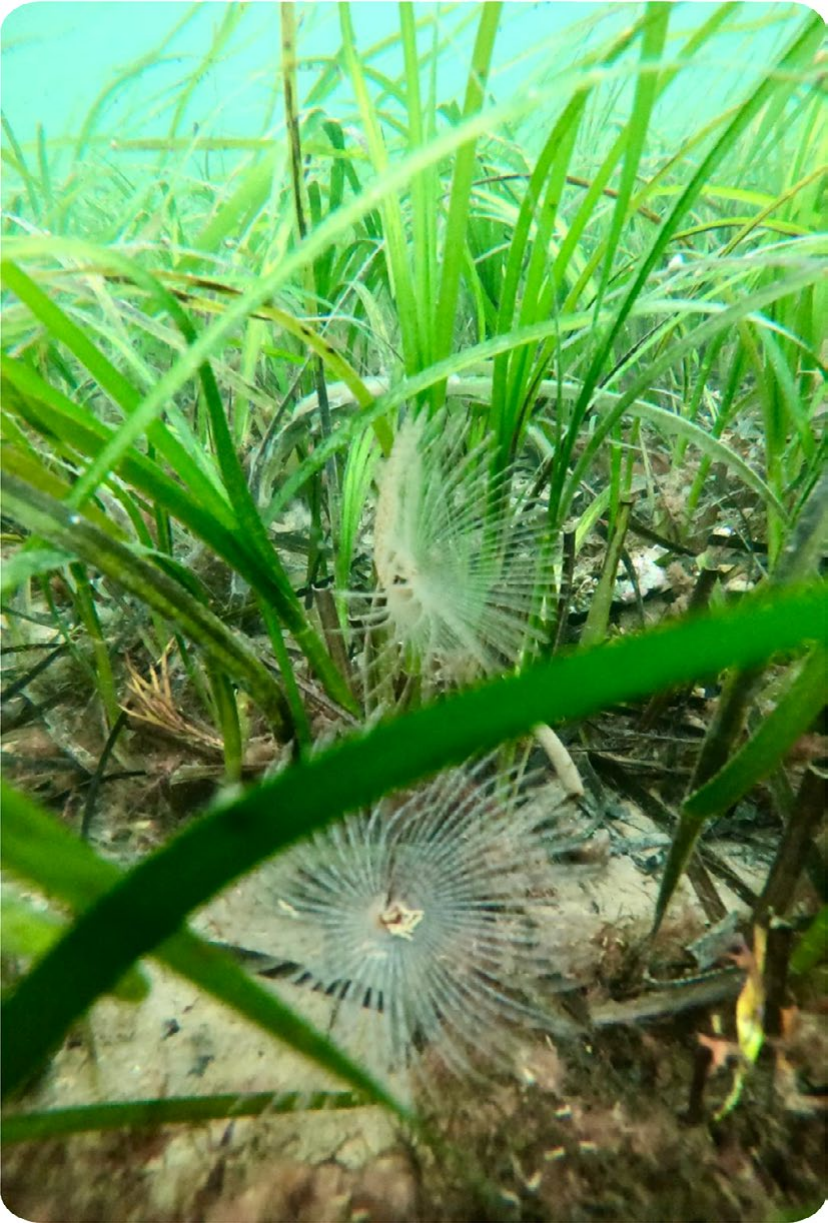
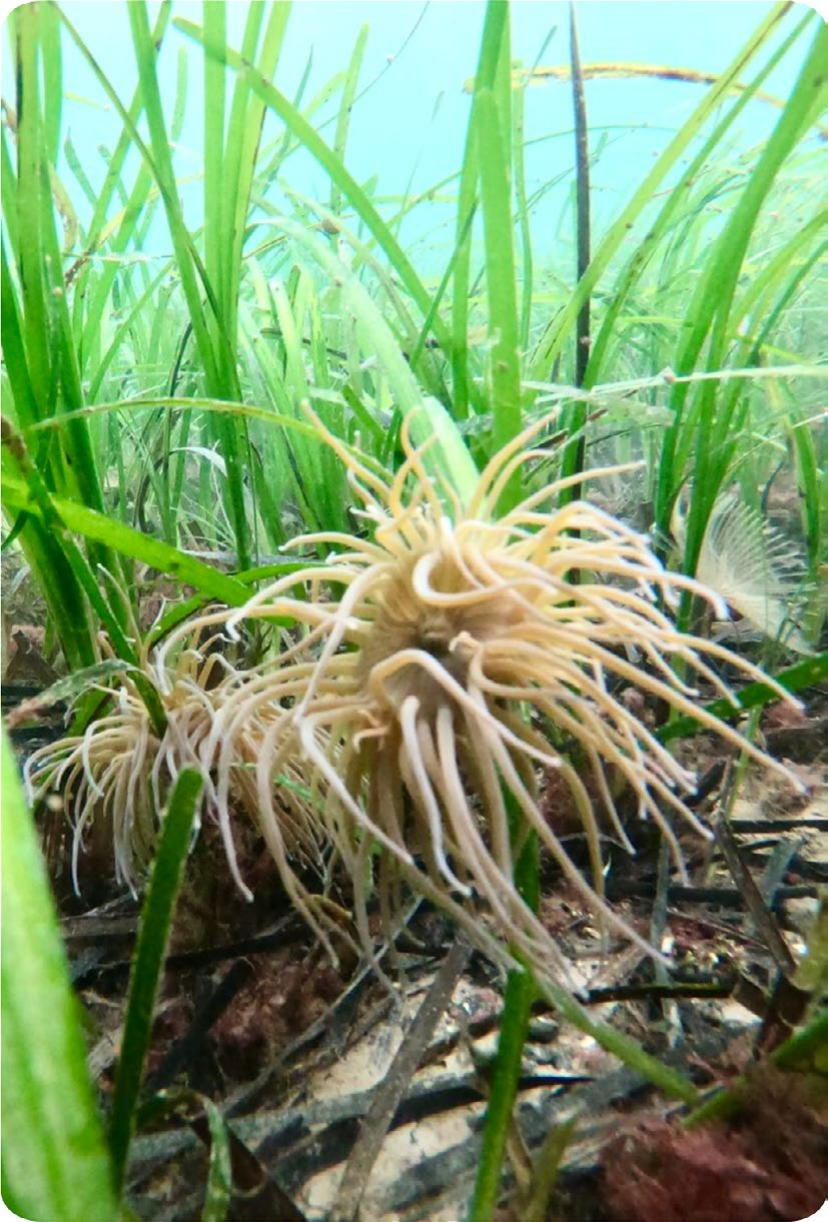	The significance of seagrass habitats to ecosystem services and function has led to concerted efforts to restore seagrass habitats (Orth et al., 2020; Paulo et al., 2019; Tan et al., 2020). In relation to this, experiments have shown the importance of genetic diversity in supporting the successful re‐establishment of seagrass populations following transplantation. Increased genetic diversity has been associated with increased survival, biomass, plant density, and a range of ecosystem services including faunal abundance, nutrient retention, and net primary productivity following transplantation for restoration purposes (Evans et al., 2018; Reynolds et al., 2012). These findings, along with evidence of the stabilizing role of increased genetic diversity under stress in seagrass systems (Evans et al., 2017; Plaisted et al., 2020; Ehlers et al., 2008; Reusch et al., 2005; DuBois et al., 2021), highlight the importance of considering genetic diversity and genetic assessment in the restoration of coastal marine habitats (Mijangos et al., 2015; Wood et al., 2020).

However, the evolutionary and ecological effects of genetic diversity are not always clear‐cut. Variation in the effects of genetic diversity on ecosystem function and community composition have been observed between species, on different aspects of community assemblage, under various levels of stress, and over different spatial and community scales (Barantal et al., 2019; Bongers et al., 2020; Chalmandrier et al., 2017; DuBois et al., 2021; Raffard et al., 2019). Furthermore, epigenetic influences from variation in the present‐ and past‐generation environments have also been found to add to functional diversity within species, complicating the estimation of contributions from genetic diversity to ecological function and adaptation (Bogan et al., 2020; Nguyen et al., 2020; Puy et al., 2020). Studies have also pointed out variation in the significance of genetic diversity to evolutionary potential between organism groups, with numerous examples of species or populations persisting over time despite low levels of genetic diversity (Attard et al., 2015; Morin et al., 2020). Differences in mutation rate can have a significant effect on the prevalence of beneficial mutations occurring within a population, along with differences in effective population size (Rousselle et al., 2020). Crucially, the strength and direction of past and contemporary selection on functional genetic diversity can have a much greater influence on functional adaptation and short‐term evolutionary potential than longer‐term changes in neutral genetic diversity (Teixeira & Huber, 2021).

## 3 IMPACTS ON GENETIC DIVERSITY IN THE MARINE ENVIRONMENT

Despite the importance of biodiversity to planetary and societal health, strategic plans and government initiatives have so far failed to halt biodiversity loss and ecosystem degradation around the world (IPBES, 2019). Increased anthropogenic pressures of pollution, urbanization, and exploitation have led to widespread declines in marine biodiversity and habitat condition and structure over the last century (Bugnot et al., 2021; Díaz et al., 2019; Jouffray et al., 2020; Pauly & Zeller, 2016). In addition, the intensifying effects of global climate change have driven changes in population distributions, pushing certain species to their physiological limits, and increasing the risks of extinction in many species at local and global scales (Babcock et al., 2019; Burrows et al., 2011; Simon‐Nutbrown et al., 2020; Wilson et al., 2019).

Increasing anthropogenic and climate pressures are likewise resulting in significant declines in the genetic diversity of wild populations, largely driven by human influences, including overharvesting, habitat loss and fragmentation, genetic introgression from invasive and domesticated species, and climate change (Allendorf et al., 2008; Leigh et al., 2019; Mimura et al., 2017; Miraldo et al., 2016). Impacts on genetic diversity can have delayed responses, often taking several generations to become apparent following the initiation of population decline (Aavik et al., 2019; Berger‐Tal & Saltz, 2019; Gurgel et al., 2020). While populations may expand rapidly once limiting factors or pressures have been removed, the replacement of genetic variation through mutation is a much slower process (Frankham et al., 2014; Rousselle et al., 2020). A meta‐analysis of 30 pinniped species, for instance, highlighted lasting signals of genetic bottlenecks in as many as a third of the species studied, primarily as a result of commercial exploitation during the 18th and 19th centuries (Stoffel et al., 2018). Losses of genetic diversity can thereby leave long‐lasting effects on genetic diversity and functional variation within populations, in turn affecting long‐term resilience, function, and adaptive capacity (Kess et al., 2019; Stoffel et al., 2018; Takahashi et al., 2016).

**FIGURE 1:**
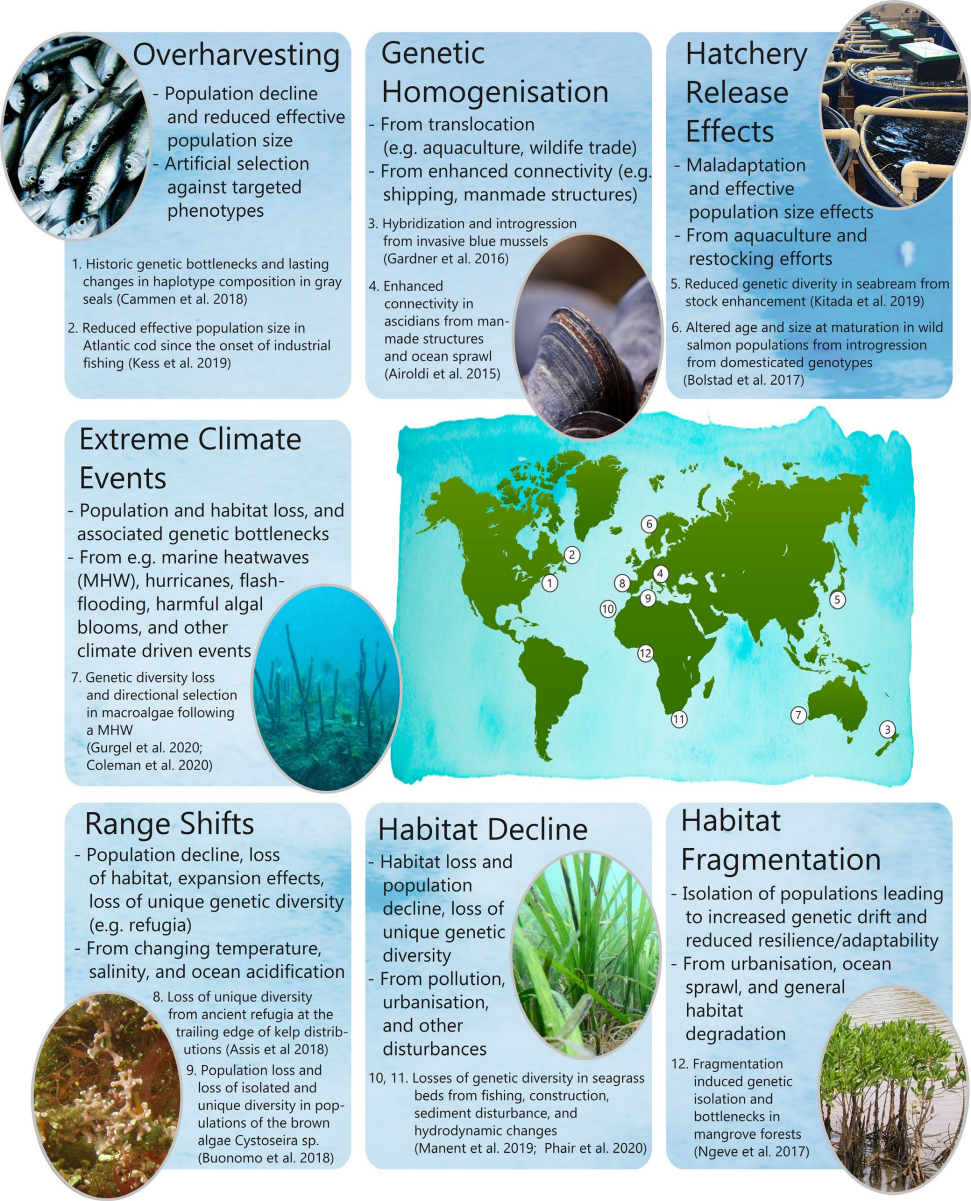
Conceptual representation of the principal causes of loss of genetic diversity in the marine environment, including relevant examples from around the world. (Photo credits: Alasdair O'Dell, Melinda Coleman, Wikipedia Creative Commons)

In the marine environment, anthropogenic impacts have affected genetic diversity in wild populations in numerous ways (Figure 1). Fishing pressures and the selection of certain phenotypes have led to fisheries‐induced evolutionary effects such as reduced body size and maturation at smaller body sizes, as well as reductions in genetic diversity through population declines and overharvesting (Heino et al., 2015; Lim et al., 2021; Pinsky & Palumbi, 2014; Price et al., 2019). Evidence of genetic bottlenecks and reductions in effective population size have been identified in Atlantic cod populations (*Gadus morhua*) from the North‐West Atlantic, in Pacific Salmon from British Columbia, as well as in populations of New Zealand snapper (*Pagrus auratus*) following overexploitation from industrial fishing practices (Hauser et al., 2002; Kess et al., 2019; Price et al., 2019). Notably, reductions of effective population size and losses of genetic diversity were observed within functional regions of the genome associated with migration behavior in Atlantic cod (*Gadus morhua*), highlighting the potential for overexploitation to affect functional genetic variation and potentially vital adaptive behaviors (Kess et al., 2019).

Pollution and habitat loss have likewise led to losses of genetic diversity in key primary producers and foundation species through population declines and isolation effects (Bryan‐Brown et al., 2020; de los Santos et al., 2019). Increasing urbanization and pollution in coastal areas for instance has led to the degradation of intertidal and subtidal habitats, including saltmarsh, seagrass, and mangrove‐dominated communities (Bryan‐Brown et al., 2020; Gu et al., 2018; Krause‐Jensen et al., 2021). Losses of susceptible genotypes, as well as local populations, can lead to declines in genetic diversity from habitat degradation. Activity surrounding the development of a port in Gran Canaria (Spain), for example, was associated with a 33% decrease in genetic diversity (estimated as observed heterozygosity) in nearby seagrass populations over a twelve‐year period, a pattern that was not observed in undisturbed control sites (Manent et al., 2020). Other coastal activities, including fishing, leisure boating, mining, and changes in estuarine flow regimes, have similarly been associated with decreased genetic diversity in affected seagrass populations (Alotaibi et al., 2019; Phair et al., 2020). In addition, the fragmentation of marine habitats, such as that seen in coastal mangrove and seagrass communities, can also affect genetic diversity through isolation effects and subsequent effects of inbreeding and increased genetic drift, limiting the ability of populations to migrate or genetically adapt, and thereby elevating the risks of maladaptation and local extinction (Binks et al., 2019; González et al., 2020; Toczydlowski & Waller, 2019).

Unprecedented rates of species introductions and pest invasions in the marine environment, alongside accidental and deliberate releases from hatchery environments, have been another major contributor to genetic diversity loss in native biota due to competition, predation, infection, or introgression effects (Glover et al., 2017; Laikre et al., 2010; Olden et al., 2004; Teagle & Smale, 2018). The translocation of non‐native species and populations in the shellfish aquaculture industry for instance has resulted in widespread hybridization, impacting the genetic diversity of natural wild populations, as well as impacting the physiology of farmed populations (Gardner et al., 2016; Michalek et al., 2016; Šegvić‐Bubić et al., 2020; Varney et al., 2018). Aquaculture escapees, as well as hatchery‐bred populations released for marine stock enhancement projects, have likewise had widespread effects on the genetics of wild populations, including changes in allele frequencies and population structure, hybridization and introgression, and loss of genetic diversity (Glover et al., 2017; Kitada, 2018).

The effects of climate change have also been felt keenly in the marine environment. Rising ocean temperatures, increasing acidification, and changing ocean currents are contributing to fundamental and irreversible ecological transformations in marine ecosystems at a global scale (Babcock et al., 2019; Harris et al., 2018; Hoegh‐Guldberg & Bruno, 2010). Major losses of genetic diversity have been linked to population declines resulting from both extreme weather events such as marine heatwaves, as well as from more gradual environmental changes, and such events are expected to increase in frequency in the future (Buonomo et al., 2018; Gurgel et al., 2020; Oliver et al., 2019; Simon‐Nutbrown et al., 2020; Wernberg et al., 2018). Extreme climate events in the marine environment, such as heatwaves, flash flooding, and chronic stress effects, have already been shown to impact genetic diversity through population losses and bottlenecks, as well as through intensive selection processes (Coleman, Minne, et al., 2020; Coleman, Wood, et al., 2020; Griffiths et al., 2020; Gurgel et al., 2020; Vincenzi et al., 2017). Critically, the impacts of climate stress on many foundation species, such as corals and marine macrophytes, may also impact genetic diversity at the community level through cascading ecological effects (Barantal et al., 2019; Blanchet et al., 2020; Koricheva & Hayes, 2018). However, genetic impacts from acute and chronic climate stress can vary between organisms, as well as between populations spanning species ranges, depending on differences in selection history, adaptive traits, and stress thresholds (Duarte et al., 2018; Miller et al., 2020; Pilczynska et al., 2016; Straub et al., 2019). In addition, extreme climatic events, such as bleaching events in corals, and marine heatwaves in macroalgae, have been highlighted as potentially significant drivers of directional selection and offer important sources of resilient genotypes that can contribute to the success of restoration and assisted evolution programs (Coleman, Minne, et al., 2020; Coleman, Wood, et al., 2020; Coleman & Wernberg, 2020; Morikawa & Palumbi, 2019; Wood, Marzinelli, et al., 2021).

Range shifts in species and populations, driven by climate change and associated shifts in the physical ocean climate, are being increasingly reported in marine ecosystems (Bashevkin et al., 2020; Griffith et al., 2018; Vergés et al., 2014; Wilson et al., 2016). Range shifts pose a significant risk to genetic diversity in wild populations through new biotic interactions and shifts in trophic networks, population declines and bottlenecks, and the local extinctions of unique pools of genetic diversity (Garnier & Lewis, 2016; King et al., 2017; Pauls et al., 2013; Wróblewska & Mirski, 2018). In the marine environment, shifts in temperature, salinity, and ocean acidity are expected to result in population range shifts and subsequent declines in genetic diversity in a wide range of organisms (Buonomo et al., 2018; Donelson et al., 2019; Johannesson et al., 2020; Simon‐Nutbrown et al., 2020; Wilson et al., 2019). In particular, the loss of trailing edge and ancient refugia populations, which can represent important sources of unique genetic diversity, has been highlighted as a potential threat to a number of marine species groups, including cold‐temperate macroalgae and invertebrates (Assis et al., 2018; Hampe & Petit, 2005; Scheider, 2018). Depending on local climate velocities and species traits, it is expected that selection will be unable to keep pace with rapid climate change and that interventions such as assisted migration and gene flow will be needed to preserve patterns of endemism and adaptive potential in wild populations (Capblancq et al., 2020; Coleman & Wernberg, 2020; Duarte et al., 2018; Hoffmann et al., 2021; Novak et al., 2020; Wood, Marzinelli, et al., 2021).

## 4 THE ROLE OF GENETIC DIVERSITY IN THE UN DECADE OF OCEAN SCIENCE

The management and enhancement of biodiversity lie at the heart of the Decade's objectives of a healthy and productive ocean (Tables 1 and 2). Maintaining resilience and adaptive capacity in marine ecosystems through the maintenance of genetic diversity will be a central component in this. Aspects of genetic diversity underpin a wide range of ecologically and socioeconomically important factors in the marine environment which are integral to the central aims of the Decade Implementation Plan (Table 3). Habitat restoration, assisted evolution, and spatial marine planning, as well as the improved management of fisheries and the aquaculture industry, will play important roles in achieving the objectives of the Decade program (Janssen et al., 2017; Khoury et al., 2015; Miller et al., 2020; Novak et al., 2020; Waltham et al., 2020; Xuereb et al., 2020). Each of these approaches, in turn, is supported and facilitated by the understanding and maintenance of genetic diversity (Bernatchez et al., 2017; Houston et al., 2020; Wood et al., 2020). Conversely, losses of genetic diversity due to direct anthropogenic activities and environmental changes can have significant socioeconomic consequences for marine activities, as well as compromising the long‐term provision of ecosystem services imparted by marine habitats and biomes (Bernatchez et al., 2017; Blasiak et al., 2020; Stange et al., 2020).

**TABLE 1:** Overview of the Decade Outcomes (OUs) describing the desired state of the ocean, and of society's sustainable interaction with the ocean, at the end of the Decade

Ocean Decade Outcomes	
OU1	A clean ocean where sources of pollution are identified, reduced, or removed.
OU2	A healthy and resilient ocean where marine ecosystems are understood and managed.
OU3	A productive ocean supporting sustainable food supply and a sustainable ocean economy
OU4	A predicted ocean where society understands and can respond to changing ocean conditions
OU5	A safe ocean where life and livelihoods are protected from ocean‐related hazards
OU6	An accessible ocean with open and equitable access to data, information, technology, and innovation.
OU7	An inspiring and engaging ocean where society understands and values the ocean in relation to human well‐being and sustainable development.

**TABLE 2:** Overview of the Decade Challenges (CHs)—the highest level of the Decade Action Framework. These represent the most immediate and pressing priorities that can be translated into meaningful action, both globally and locally

Ocean Decade Challenges	
CH1	Understand and map land and sea‐based sources of pollutants and contaminants and their potential impacts on human health and ocean ecosystems, and develop solutions to mitigate or remove them.
CH2	Understand the effects of multiple stressors on ocean ecosystems and develop solutions to protect, monitor, manage, and restore ecosystems and their biodiversity under changing environmental conditions, including climate.
CH3	Generate knowledge, support innovation, and develop solutions to optimize the role of the ocean to contribute to sustainably feeding the world's population under changing environmental and social conditions.
CH4	Generate knowledge, support innovation, and develop solutions to contribute to equitable and sustainable development of the ocean economy under changing environmental and social conditions.
CH5	Enhance understanding of the ocean‐climate nexus and use this understanding to generate solutions to mitigate, adapt, and build resilience to the effects of climate change, and to improve services including improved predictions and forecasts for weather, climate, and the ocean.
CH6	Expand multi‐hazard warning systems for all biological, geophysical, and weather and climate‐related ocean hazards, and mainstream community preparedness and resilience.
CH7	Ensure a sustainable ocean observing system that delivers timely data and information accessible to all users on the state of the ocean across all ocean basins.
CH8	Develop a comprehensive digital representation of the ocean, including a dynamic ocean map, through multi‐stakeholder collaboration that provides free and open access to explore, discover, and visualize past, current, and future ocean conditions.
CH9	Ensure comprehensive capacity development and equitable access to data, information, knowledge, and technology across all aspects of ocean science and for all stakeholders regardless of geography, gender, culture, or age.
CH10	Ensure that the multiple values of the ocean for human well‐being, culture, and sustainable development are recognized and widely understood, and identify and overcome barriers to the behavior change that is required for a step change in humanity's relationship with the ocean.

**TABLE 3:** Overview of the importance of genetic diversity and its maintenance and monitoring to key ocean topics, and their relevance to the central Outcomes and Challenges of the Decade of Ocean Science Implementation Plan (see Tables 1 and 2)

Theme/Topic	Relevance of Genetic Diversity	Relevance to the Decade Outcomes (OU) and Challenges (CH)
Marine spatial management and conservation planning	Connectivity assessments between protected areas	OU 2
	Prioritization of populations—for example, isolated populations, refugia, and diversity hotspots	OU 2
	Assessments and prioritization of putatively adaptive variation	OU 2
Habitat restoration	Facilitating translocation and restoration success through increased resilience	OU 2, CH 2
	Assessment of genetic diversity of restoration efforts—avoiding genetic impacts from poor genetic make‐up	OU 1 and 2, CH 1 and 2
	Identifying putatively beneficial genotypes and functional markers to improve long‐term resilience and adaptive success of restoration efforts	OU 2, CH 2
Assisted evolution	Identifying isolated populations potentially at risk without assisted evolution or demographic rescue	OU 2, CH 2, 3, and 5
	Identifying putatively beneficial genotypes and functional markers to improve long‐term resilience through assisted migration, translocation, or breeding	OU 2, CH 2
	Assessing potential effects of maladaptation and genetic pollution from assisted evolution efforts	OU 1, CH 1
Genebanking and ex situ conservation	Assessment and maintenance of genetic diversity and effective population size in ex situ conservation efforts	OU 1
	Underpinning genebanking and biobanking efforts to support ex situ preservation of biodiversity for conservation and breeding	OU 2 and 3, CH 2, 3 and 4
Aquaculture management, breeding and monitoring	Assessment and maintenance of genetic diversity and effective population size in breeding efforts	OU 3, CH 3
	Monitoring of aquaculture impacts on wild‐relative populations—for example, introgression and hybridization effects	OU 1 and 3, CH 1 and 3
	Management of translocation	OU 1 and 3, CH 1 and 3
	Preservation and maintenance of genetic diversity in wild populations and biobanking or genebanking programs for long‐term breeding and diversification	OU 3, CH 3 and 4
	Advanced breeding programs based on functional genetic markers	OU 3, CH 3 and 5
Fisheries management and monitoring	Identification of fisheries management units and populations	OU 2 and 3, CH 2 and 3
	Assessments of the genetic and evolutionary impacts of overharvesting	OU 2 and 3, CH 2 and 3
	Assessment of evolutionary trajectories of fisheries under climate change	OU 2, 3, and 4, CH 2 and 3
Bioprospecting and Marine Genetic Resources	Identification of novel marine genetic resources for biotechnological and pharmaceutical products	OU 3 and 7, CH 3, 4, and 7
	Policy, patenting, and management of genetic resources from the marine environment	OU 3, 6, and 7, CH 4 and 10
Monitoring of anthropogenic impacts and climate change	Assessments of genetic impacts and effects from climate change including extreme climate events and longer‐term range shifts	OU 2 and 4, CH 2 and 8
	Assessments of genetic impacts from anthropogenic activities, including marine urbanization, resource extraction and exploitation, pollution, and globalization.	OU 1, 2, 3, and 4, CH 1, 2, and 3
Wildlife crime and trade	Monitoring tools for forensic wildlife crime prevention—for instance the detection, identification, and sourcing of protected species and populations in marine animal products, food types, and medicines	OU 3 and 6, CH 2, 3, and 4

Ecosystem restoration is forecast to play a prominent role in the enhancement of coastal and marine environments in the next ten years, driven in part by the parallel declaration of the UN Decade on Ecosystem Restoration for 2021–2030 (Bayraktarov et al., 2020; Stewart‐Sinclair et al., 2020; UNEP/FAO, 2020; Waltham et al., 2020). Though its inclusion remains patchy, the use of genetic data in ecosystem restoration efforts is becoming more widespread (Breed et al., 2013; Mijangos et al., 2015; Wood et al., 2020). The consideration of genetic diversity, as well as associated factors such as connectivity and effective population size, should be fundamental to the implementation of restoration efforts, increasing the chances of longer‐term restoration success and population resilience (Mijangos et al., 2015). As an example, the maintenance of genetic diversity in seagrass restoration efforts has been shown to support successful long‐term re‐establishment through increased environmental resilience and complementarity effects (Evans et al., 2018; Reynolds et al., 2012) (Box 1). Genetic approaches also represent important management tools for the identification of key locations for restoration, as well as suitable donor populations, by providing estimates of genetic diversity, connectivity, and demographic parameters (Jahnke et al., 2020). Advances in genomic techniques are also allowing the identification of resilient “climate‐ready” genotypes or functional markers from donor populations for enhancing the resilience of restored populations (Carvalho et al., 2021; Coleman, Minne, et al., 2020; Coleman, Wood, et al., 2020; Coleman & Wernberg, 2020; Connolly et al., 2018; Wood, Marzinelli, et al., 2021).

Genetic monitoring tools will likewise play a fundamental role in the sustainable management of ocean fisheries and aquaculture efforts. Estimates of wild fisheries and aquaculture production suggest that up to 19% of the global demand for meat by 2050 may be from the sea (Costello et al., 2020). However, the sustainability of that production is strongly reliant on the effective management of wild fisheries stocks (Hilborn et al., 2020). Genetic data can improve the effectiveness of management efforts by providing important insights into population size, demography, and structure in wild fisheries (Bernatchez et al., 2017; Papa et al., 2020). In particular, genetic data have provided key information for identifying mismatches between existing fisheries management units and biologically relevant population units, as in the cases for African yellowfin tuna (*Thunnus albacares*) and Atlantic cod (*Gadus morhua*) (Johansen et al., 2020; Mullins et al., 2018). Impacts on genetic diversity and evolutionary trajectories in marine fisheries have also been significant and can affect not only the quality of fisheries harvests in terms of body size but also the sustainability of fisheries as populations face changes in their environment with reduced genetic diversity and capacity for adaptation (Bernatchez, 2016; Kess et al., 2019; Pinsky & Palumbi, 2014).

The contribution of aquaculture to global food production is forecast to increase at a much greater rate than wild fisheries, with up to an estimated 44% of food from the sea being produced by aquaculture by 2050 (Costello et al., 2020). Once again, genetic data and genomic tools will be fundamental to the advancement of breeding and selection in farmed species, as well as for impact monitoring from aquaculture practices and the informed management of genetic resources in farmed and wild‐relative populations (Graf et al., 2021; Houston et al., 2020). In terrestrial farming and agriculture, the use of genetic diversity from crop wild relatives (CWR) to improve and diversify existing crops is becoming increasingly important in developing sustainable and climate‐resilient breeds (Brozynska et al., 2016; Dempewolf et al., 2017; Engles & Thormann, 2020). The use of genetic monoculture has already proven a risk to sustainable production in aquaculture sectors, such as seaweeds and shrimp, due to the global proliferation of pathogens and diseases, and the susceptibility of monocultures to environmental change (Cottier‐Cook et al., 2016; Stentiford et al., 2017). The loss of genetic diversity from wild relatives therefore represents a loss of potential functional diversity for the breeding and diversification of climate and disease resilient strains in aquaculture in the future (FAO, 2019a; Goecke et al., 2020; Lind et al., 2012; Wade et al., 2020).

A loss of genetic diversity in the marine environment also represents the loss of potential bioprospecting discoveries, including genetic information to develop products for pharmacological and biotechnological use (Arnaud‐Haond et al., 2011; Arrieta et al., 2010; Blasiak et al., 2018, 2020; Rabone et al., 2019; Sigwart et al., 2020). Species within the marine phyla of the Porifera (sponges) and Cnidaria (including jellyfish, corals, and anemones), for instance, have already contributed over 7500 novel marine natural products in recent decades, including potent cancer‐treatment drugs, antibacterial products, and novel fluorescent proteins for biotechnological and medical research (Leal et al., 2012; Mehbub et al., 2014; Rocha et al., 2011). Though efforts have so far focused on the exploration of species biodiversity in relation to novel marine bio‐products, variation within species, determined by genetic diversity, may also prove a rich source of novel products in the future.

The improved management and protection of marine ecosystems, through spatial conservation planning and impact monitoring, will be central to achieving the clean, resilient, and productive ocean envisioned in the Decade Outcomes (Tittensor et al., 2019; Wilson et al., 2020). Once more, the monitoring and maintenance of genetic diversity will be fundamental in supporting these outcomes. Genetic and genomic data are being integrated more widely into the design and implementation of marine spatial conservation planning (Beger et al., 2014; Coleman et al., 2017; Nielsen et al., 2020; Xuereb et al., 2019). In particular, genetic data can offer important estimates of connectivity among networks of marine protected areas, a central driver of resilience and adaptability over wider management regions (Jenkins & Stevens, 2018; Xuereb et al., 2019). In addition, information obtained from adaptive genetic markers can offer further insights into functional and adaptive variation among populations, and can allow the incorporation of information on adaptation into the planning and prioritization of management efforts (Miller et al., 2020; Waldvogel et al., 2020; Wilson et al., 2020; Xuereb et al., 2020). In coral reef conservation, for example, the identification of putatively heat‐stress‐adapted genotypes in certain reefs, combined with estimates of connectivity between those reefs and nonadapted populations, provided an informed framework for the effective prioritization of management and conservation efforts in the region (Selmoni et al., 2020).

The importance of biodiversity to cultural values has been emphasized by the inclusion of cultural context as a universal factor in Nature's Contributions to People (NCP) (IPBES, 2019). Genetic diversity has likewise been shown to support and contribute to numerous NCPs of cultural importance (Stange et al., 2020). Traditional fishing practices in First Nations communities have been found to be significantly influenced by Pacific salmon's behavioral variation associated with genetic diversity in the Fraser River (Nesbitt & Moore, 2016). Changes in the timing and intensity of seasonal salmon runs have been directly linked to the erosion of genetic diversity in key determinant genes (Thompson et al., 2019). Such changes can have knock‐on effects to the sustenance and livelihoods of those communities that rely on Pacific salmon at certain times of year, in turn affecting factors of cultural identity and sense of place (Moncrieff, 2017; Oke et al., 2020). The protection and maintenance of genetic diversity in the marine environment are, therefore, highly relevant to the aims and objectives of the Decade in supporting the sustainable use of the marine environment for societal needs and welfare.

## 5 IMPROVING OUR UNDERSTANDING OF GENETIC DIVERSITY IN THE DECADE OF OCEAN SCIENCE

The principle objectives of the Decade affirm the need to better understand our oceans and to improve the interface between science and policy in order to support their sustainable management (Table 4) (IOC, 2019). Understanding the role of genetic diversity in influencing species adaptation and ecosystem resilience and function, and how environmental pressures can impact spatial and temporal patterns of genetic diversity, will be of key importance in the effective management and protection of the marine environment.

**TABLE 4:** Overview of the Decade Objectives which focus on key processes to ensure the successful realization of the Decade Outcomes and Challenges

Ocean Decade Objectives	
OB1	Increase capacity to generate, understand, manage, and use ocean knowledge
OB2	Identify and generate required ocean data, information, and knowledge
OB3	Build comprehensive understanding of the ocean and ocean governance systems
OB4	Increase the use of ocean knowledge

Rates of change in the marine environment underline the urgent need for increased monitoring and baseline surveying of genetic diversity in the coming decade (Burrows et al., 2011; Hoban, Bruford, et al., 2020; Hoban, Campbell, et al., 2020). The breadth of understudied ecosystems in the ocean, as well as the imperceptible nature of genetic diversity and the cryptic influences of many anthropogenic effects on it, suggest that many of these changes occur undocumented or unnoticed in the marine environment (Berger‐Tal & Saltz, 2019; Gurgel et al., 2020; Kennedy et al., 2019; Taylor & Roterman, 2017). Efforts on genetic monitoring and management may also not correspond to those regions and ecosystems most at risk of losing genetic diversity, further deepening inequalities in the ability to access genetic resources and enhance the provision of ecosystem services and benefits (Blasiak et al., 2020). The failure to document genetic diversity and monitor its changes in all seas and oceans thereby risks the irreversible loss of potential genetic resources for human use, as well as the danger of false perceptions of baseline genetic diversity in the marine environment at future points in time (Soga & Gaston, 2018; Mihoub et al., 2017; Blasiak et al., 2020; Coleman, Minne, et al., 2020; Coleman, Wood, et al., 2020).

Crucially, our understanding of changes in genetic diversity will rely on improved temporal sampling and surveying (Hoban et al., 2014; Mihoub et al., 2017). Temporal sampling of genetic diversity remains scarce for many organisms, though efforts are being made to implement genetic monitoring in management and conservation plans (Reynolds et al., 2017). For example, genetic monitoring of Atlantic cod populations in Norway has offered important insights into the effectiveness of coastal Marine Protected Areas (MPAs) for separate stock populations, with direct consequences on the management of commercial harvesting in the region (Johansen et al., 2018). The stochastic nature of dispersal and recruitment in the marine environment can add additional difficulties to understanding temporal changes in population genetics, further emphasizing the need for longer‐term sampling series (Riginos et al., 2016; Siegel et al., 2008). Efforts should therefore be made to increase funding for longer‐term monitoring and the development of genetic time‐series, as well as encouraging the sequencing and analysis of suitable historic samples were available (Cammen et al., 2018; Hoban et al., 2014; Price et al., 2019).

## 6 FUTURE PERSPECTIVES ON ADAPTATION IN THE OCEAN

Developing our understanding of the role and significance of genetic diversity in adaptation will also enhance the management and maintenance of ocean biodiversity (Duarte et al., 2018; Wilson et al., 2020). While the role of functional genetic diversity in long‐term adaptive evolution is undisputed, many questions remain about the capacity for selection to keep pace with rapid changes in the environment from anthropogenic climate change (Capblancq et al., 2020; Duarte et al., 2018). Understanding temporal and spatial limitations on genetic adaptation will be key in determining the potential for adaptive responses in populations under threat, and identifying whether more active conservation interventions such as assisted gene flow or assisted evolution may be required (Coleman & Goold, 2019; Gaitán‐Espitia & Hobday, 2021; Hoffmann et al., 2021; Novak et al., 2020). The significance of genetic diversity to adaptation in comparison with epigenetic mechanisms and the influence of organism microbiomes also remains an important question with direct consequences for marine management and conservation (Duarte et al., 2018; Nguyen et al., 2020; Epstein et al., 2019; Voolstra & Ziegler, 2020; Liew et al., 2020). Finally, a greater theoretical understanding of the mechanisms underlying functional genetic variation and adaptation, including the roles of larger structural variants, epistasis and polygenic effects, and the interaction between genetic and epigenetic functions, will also be critical in understanding and applying genetic data to maximum effect (Duarte et al., 2018; Teixeira & Huber, 2021; Wellenreuther & Hansson, 2016; Wellenreuther et al., 2019).

Improvements in the understanding of adaptation and its mechanisms will benefit every aspect of ocean management and health, from the prioritization of conservation efforts, through to advanced fisheries management, and the development of climate‐resilient aquaculture and ecosystem restoration programs (Bernatchez et al., 2017; Houston et al., 2020; Waldvogel et al., 2020; Xuereb et al., 2020). The importance of these questions in understanding ocean ecosystems exemplifies the core message of the Decade of strengthening “the science we need for the ocean we want.”

## 7 OCEAN GENETIC DIVERSITY IN INTERNATIONAL FRAMEWORKS

Global recognition of the significance of genetic diversity to ecosystem functionality, resilience, and evolutionary potential is increasing (Stange et al., 2020). This has been emphasized by the direct inclusion of the maintenance of genetic diversity as a primary goal for the 2050 “Vision of Biodiversity” in the recently updated Zero Draft of the Post‐2020 Global Biodiversity Framework (CBD, 2020a,2020b). However, responses to the Zero Draft have pointed out the weaknesses and lack of clarity surrounding the goals of this framework for the maintenance and protection of genetic diversity, in particular the lack of distinct Action Targets addressing the maintenance of genetic diversity by 2030, as well as the need for suitable indicators for the monitoring of genetic diversity in wild populations (Hoban, Bruford, et al., 2020; Hoban, Campbell, et al., 2020; Laikre et al., 2020).

In the marine environment, the importance of genetic diversity to biodiversity and Nature's Contributions to People (NCP) has likewise been widely acknowledged, in particular through the work of groups such as the High Level Ocean Panel and the FAO (Blasiak et al., 2020; FAO, 2021; Stuchtey et al., 2020). Nevertheless, genetic diversity and its maintenance remain overlooked in other international programs and frameworks, including in the Decade Implementation Plan (IOC, 2020). The importance of protecting and maintaining marine biodiversity is fully acknowledged by the Decade; however, failing to explicitly consider all three levels of biodiversity may lead to genetic diversity being overlooked in associated policy, governance, and action (Hoban, Bruford, et al., 2020; Hoban, Campbell, et al., 2020; Laikre, 2010).

The Decade Implementation Plan does emphasize the importance of fair and equitable access to marine resources, knowledge, and technology, and genetic resources are directly mentioned in this context (IOC, 2020; Österblom et al., 2020). The Nagoya Protocol has given recognition and a legal framework for fair and equitable access to genetic resources and the benefits arising from their utilization since 2014 (CBD, 2011; Smith et al., 2018), and there are ongoing negotiations to extend the application of these principles to marine areas beyond national jurisdiction (BBNJ) (Blasiak et al., 2020; Santo et al., 2020; Rabone et al., 2019). The Decade is well placed to support these frameworks through contributions to data sharing, expertise, and capacity building. Such efforts will be strongly dependent on the improved monitoring and understanding of genetic diversity in the marine environment.

## 8 GENETIC DIVERSITY AT THE SCIENCE–POLICY INTERFACE

Communicating the importance of genetic diversity to policymakers, regional managers, and wider stakeholders of the marine environment will facilitate the advancement of our understanding of genetic diversity, as well as allowing that knowledge to be applied more effectively in the management of the marine environment and its development for human use (Pérez‐Espona & ConGRESS Consortium, 2017).

Effective indicators of genetic diversity, such as those proposed for the recent Zero Draft of the Post‐2020 Global Biodiversity Framework, are instrumental in communicating the status of genetic diversity to management, governance, and legislators (CBD, 2020a,2020b; Hoban, Bruford, et al., 2020; Hoban, Campbell, et al., 2020). Effective targets at a national level confer a degree of government accountability and facilitate wider compliance in monitoring and maintaining biodiversity (Xu et al., 2021). However, the effective investment and setting of national targets remain inconsistent. A recent analysis of the use of genetic diversity indicators at a national level showed that despite improvements in the inclusion and consideration of genetic diversity in biodiversity reporting, nondomesticated species continue to be overlooked, and the uptake and application of more effective genetic diversity indicators continue to lag behind other measures of biodiversity (Hoban, Bruford, et al., 2020; Hoban, Campbell, et al., 2020). Improvements in the representation of wild species and species of lower socioeconomic value in genetic diversity monitoring, as well as the wider application of effective indicator approaches, should therefore be a priority area for the management of marine genetic diversity in the decade to come (Hoban, Bruford, et al., 2020; Hoban, Campbell, et al., 2020). Additional indicator measures specific to the characteristics of marine populations and the ocean environment may also be beneficial to the management of marine genetic diversity, for example, developing improved effective population size indicators for large‐population‐size broadcast‐spawning species, and high connectivity species where estimators based on assumptions of isolation do not provide appropriate assessments of genetic parameters (Ryman et al., 2014, 2019). Including measures of the spatial and temporal distribution of genetic monitoring of ocean regions as indicators will help ensure the appropriate coverage of genetic assessment globally (Frankham et al., 2014; Teixeira et al., 2016). Genetic scorecards assessing the status and risks to genetic diversity in marine species may also offer an effective and accessible way of communicating individual or multispecies needs to legislators and governance, as has been demonstrated in terrestrial species (Hollingsworth et al., 2020).

More direct and continuous knowledge transfer between researchers, policymakers, and local environmental management groups will be vital for the effective long‐term inclusion of genetic diversity in marine management and planning (Hoban et al., 2013; Hughes et al., 2020; Pérez‐Espona & ConGRESS Consortium 2017; Sandström et al., 2019; Taft et al., 2020). In particular, the inclusion of genetic diversity in the planning of MPAs to protect areas of high or unique genetic diversity should be made in partnership between researchers, policymakers, managers, and local stakeholders (Blasiak et al., 2020; Brooks et al., 2015; Roberts et al., 2017; Xuereb et al., 2020). Cross‐party understanding of the importance of genetic diversity will be crucial to the success of such efforts. Case studies from conservation management programs in the Baltic Sea have demonstrated the effectiveness in the short‐term of lecture‐based and group‐based knowledge transfer programs addressing issues of genetic diversity (Lundmark et al., 2019). However, the findings also highlighted the importance of continuous interaction and knowledge sharing between researchers and conservation managers to ensure the effective application of genetic diversity measures in management policy over longer time periods (Lundmark et al., 2019; Sandström et al., 2019).

The offshore marine environment and, in particular, areas beyond national jurisdiction present a different set of challenges for biodiversity and genetic management. Industries such as offshore aquaculture, renewables, and deep‐sea mining are expected to expand rapidly in the coming decade (Jouffray et al., 2020; Klinger et al., 2017). With that growth will come the need for improved management and environmental monitoring as well as international frameworks such as the BBNJ to encourage compliance and cooperation (Santo et al., 2020; Lester et al., 2018). Genetic diversity should be an important consideration in this development and an integral part of environmental monitoring and reporting standards for offshore industries. The potential risks to genetic diversity in these regions from, among other impacts, offshore aquaculture escapees, habitat loss and fragmentation as a consequence of deep‐sea mining, and invasive species transport and facilitation from shipping and infrastructure, remain significant and largely overlooked in offshore planning and legislation (Coolen et al., 2020; Lester et al., 2018; Miller et al., 2018). The assessment and monitoring of genetic diversity are particularly pertinent given the lack of understanding of genetic diversity and connectivity in many of the species inhabiting these ecosystems (Baco et al., 2016; Howell et al., 2020; Taylor & Roterman, 2017). The assessment of genetic diversity in association with offshore activities would thereby serve a dual purpose of ensuring effective environmental monitoring, while also enhancing our understanding of genetic diversity in often inaccessible species and ecosystems.

The growth of ecosystem restoration in coastal environments also presents opportunities, as well as challenges, to integrate genetic‐informed management practices into environmental enhancement programs. Knowledge transfer between researchers and marine policy and management groups can support regional managers in setting the considerations for genetic diversity in restoration projects, for instance through improving connectivity, stipulating the need for genetic baselines of donor and recipient populations, or considering assisted evolution approaches to enhance environmental resilience in restoration efforts (Breed et al., 2019; Mijangos et al., 2015; Wood et al., 2020; Wood, Marzinelli, et al., 2021).

At a wider level, communicating the importance of genetic diversity to those funding ocean restoration may offer a more effective means of integrating genetic management practices into biodiversity enhancement projects (Vanderklift et al., 2019). Investment in blue nature capital is aimed at facilitating the transition of the ocean economy to a sustainable model whilst simultaneously enhancing ocean biodiversity and ecosystem benefits (de Vos & Hart, 2020). Investment funds such as the USD 212 million Credit Suisse Ocean Engagement Fund started in September 2020 actively engage with portfolio companies to encourage sustainable practice in the marine environment and support climate‐related and biodiversity enhancement projects (Drew et al., 2020; Tobin‐de la Puenta & Mitchell, 2020). Communicating the importance of genetic diversity to funders could stimulate uptake of better management practices for genetic diversity in funded biodiversity enhancement projects, as well as in private sector companies and industries connected to the fund. In particular, the integration of genetic diversity into standards and monitoring requirements for such funds, for instance its inclusion in stipulations of “no net loss” of biodiversity from marine activities or development, would greatly strengthen the consideration of genetic diversity as a component of biodiversity in the sustainable development of the blue economy (Niner et al., 2017, 2018).

Communicating the significance and value of genetic diversity to wider stakeholders in the marine environment, including industry, coastal user groups, and the wider public, will also support the conservation and maintenance of genetic diversity in the ocean environment (UN Global Compact, 2020; Folke et al., 2019; Österblom et al., 2020). Further communication of the significance of genetic diversity in the public sphere can contribute to a wider understanding and consideration of genetic diversity in society, as has been the case for marine plastics and global warming, and can lead to bottom‐up consumer pressures on private companies to raise their corporate social responsibility profiles (Heidbreder et al., 2019; Lindemann‐Matthies & Bose, 2008). Including aspects of genetic diversity management in third‐party certification schemes such as the Marine Stewardship Council label may also prove beneficial, as companies and industries aim to visibly and voluntarily raise their standards of environmental stewardship (Bellchambers et al., 2016; Gulbrandsen, 2009). Communicating the importance of genetic diversity to project managers involved in the development and implementation of each program is, therefore, likely to offer the most effective way of integrating genetic management into wider industry practices.

## 9 CONCLUSIONS

The Decade presents a critical opportunity to put science at the heart of ocean management in the coming decade. Scientific understanding and evidence will allow for the more effective management, protection, and sustainable development of the marine environment for human use. The maintenance of genetic diversity will play a key role in this, supporting resilience and adaptive capacity in marine ecosystems in the face of increasing environmental pressures. Improved monitoring and understanding of genetic diversity will feed directly into the improved management, protection, and development of the ocean environment and human activities within it. The Decade program provides a unique opportunity for transformative action in the monitoring and maintenance of genetic diversity, offering a vital interface between science and policy, as well as the potential for genetic data, technology, and knowledge transfer across a global network. To that aim, we suggest the following key recommendations for the advancement of genetic diversity monitoring and maintenance in the Decade:

‐ Include genetic diversity, alongside species diversity and ecosystem diversity, in the Decade vision of “the ocean we want”
‐ Test existing genetic indicators for their applicability in the oceans, and if necessary, modify them to improve their suitability
‐ Improve spatial and temporal coverage of genetic assessments, and explore the suitability of archive and museum collections for assessment over historical timeframes
‐ Include genetic diversity assessment and monitoring in ecosystem restoration best practice
‐ Recognize the importance of maintaining and protecting genetic diversity in the high seas (Areas Beyond National Jurisdiction)
‐ Include and consider marine genetic diversity in concepts of Blue Nature Capital and “no net loss” of biodiversity and encourage its inclusion in corporate social responsibility and certification schemes.
‐ Improve the monitoring of potential genetic effects from overharvesting and large‐scale hatchery and release programs in the marine environment.
‐ Improve understanding of the role of genetic diversity in adaptation and resilience, and integrate this aim in the implementation of Decade resources, including ocean observation platforms and remote sensing
‐ Support frequent and improved knowledge transfer of genetic diversity between scientists, environmental managers, policymakers, and wider stakeholders regarding genetic diversity

Crucially, the monitoring and maintenance of genetic diversity should work in synergy with other forms of environmental and biodiversity management. In doing so, management geared toward the preservation and enhancement of genetic diversity will help achieve the central vision of the Decade program of supporting ecosystem resilience and adaptability alongside the sustainable management and conservation of the ocean's resources for society's continuing needs.

## CONFLICT OF INTEREST

None declared.

## Supporting information

Supplementary Material

## Data Availability

Data sharing is not applicable to this article as no new data were created or analyzed in this study.
