# Supplementary material for: Charting a course for genetic diversity in the UN Decade of Ocean Science
Source: Evol Appl. 2021 May 4;14(6):1497–518. doi: 10.1111/eva.13224 (PMC8210796; doi:10.1111/eva.13224)
Supplement: Supplementary file 1 — Supplementary Material [file EVA-14-1497-s001.pdf]

Genetic Diversity in the UN Decade of Ocean Science Implementation Plan

Key Summary

- The second draft of the UN Decade of Ocean Science Implementation Plan overlooks the importance of genetic diversity in our oceans
- Genetic diversity is the basis for adaptation and evolutionary change at species and population levels, and is a key factor influencing the resilience and functionality of marine ecosystems
- Genetic diversity will be of key importance for maximising the potential for long term survival of marine populations and species
- Genetic diversity is expected to a play a key role in ecosystem resilience and adaptability in the face of climate change as well as supporting the sustainable development of world aquaculture, fisheries, and the Blue Economy
- Marine genetic diversity has already been severely impacted by anthropogenic influences, including overexploitation, habitat destruction, species introductions and invasions, and climate change
- Despite the importance of genetic diversity for the maintenance of biodiversity, it is still often overlooked in policy and legislation
- We believe that the Decade of the Ocean Implementation Plan should explicitly recognise the importance of genetic diversity and ensure that it is taken in to account in the next ten years
- To that end, we have made a number of suggested inclusions of genetic diversity, notably in Decade Outcome 2, Decade Outcome 3 and Decade Challenge 2
- Ultimately, the inclusion of genetic diversity will facilitate and support the maintenance of genetic biodiversity in the coming decade, and in doing so, support ecosystem resilience and adaptability in the face of climate change alongside the sustainable development of our oceans.

The Significance of Genetic Diversity

A significant omission from the second draft of the UN Decade of Ocean Science Implementation plan is the acknowledgement of intraspecific genetic diversity in relation to marine biodiversity and sustainable development [1]. Genetic diversity represents one of the three fundamental pillars of biodiversity alongside interspecies diversity and ecosystem diversity. Genetic diversity is the basis for adaptation and evolutionary change at species and population levels, and underpins the resilience and functionality of marine ecosystems [2,3]. At a community level, genetic diversity has been found to have a role in ecosystem productivity, stability, and function comparable to that of interspecific diversity [4–6]. Furthermore, changes in intraspecific genetic diversity can have intergenerational effects at population, species, and community scales and significant knock-on effects throughout trophic networks [7,8]. At an evolutionary scale, genetic diversity offers the basic units for adaptive changes that enable species to respond to changes in their environment [9,10]. Evidence suggests that adaptation is often derived from standing genetic variation in local populations and the exchange of genetic variants among populations spanning environmental gradients [11,12]. The central role of genetic diversity in adaptation and ecosystem resilience is therefore likely to be of vital importance in the face of anthropogenic climate change [13–15]. Consequently, the conservation and maintenance of genetic diversity must be a management priority for ensuring the future resilience of marine ecosystems.

Despite its importance, genetic diversity in many wild populations has significantly declined since the industrial revolution, largely driven by anthropogenic impacts including overharvesting, habitat loss and fragmentation, genetic introgression from invasive and domesticated species, and climate change [16–19]. Impacts from these can often have delayed or cryptic effects which can be easily overlooked [20,21]. Losses of genetic diversity can also take significantly longer to recover than population sizes and distribution thus leaving long lasting effects on population resilience and function [22].

In the marine environment, anthropogenic impacts have affected genetic diversity of wild populations in numerous ways. Overharvesting has led to fisheries-induced evolutionary effects, whilst pollution and habitat loss have led to losses of genetic diversity in key primary producers and foundation species [23–26]. Unprecedented rates of marine introductions and pest invasions, alongside accidental and deliberate releases from hatchery environments, have also been a major contributor to genetic diversity loss in native biota due to competition, predation, infection, or introgression effects [27–30]. The effects of climate change have also been felt more keenly in the marine environment, and genetic losses due to marine heatwaves, range shifts, and population losses are expected to increase in the next ten years if not addressed [20,31–33]. Aside from compromising environmental values, losses of genetic diversity in our seas and oceans also impact important socio-economic and cultural values. The importance of genetic diversity in fisheries, aquaculture, biotechnology and the Blue Economy is highly relevant to the coming Decade of Ocean Science and is intrinsically linked to intraspecific genetic diversity in wild populations [34–36].

Global recognition of the significance of genetic diversity to ecosystem functionality, resilience, and evolutionary potential is increasing. The recent post-2020 draft of the Convention of Biological Diversity highlights the critical role of intraspecific diversity, though early versions required major revisions to address weaknesses in initial diversity targets and indicators [37,38]. Numerous international agreements and reports have acknowledged the importance of genetic diversity in ecosystem function and resilience as well as in sustainable food and aquaculture production. These include Target 2.5 of the Sustainable Development Goals (SDGs), Targets 5 and 9 of the Global Strategy for Plant Conservation (GSPC), Target 13 of the Convention on Biological Diversity (CBD) Strategic Plan for Biodiversity 2011-2020 (https://www.cbd.int/sp/), and a number of the Food & Agriculture Organisation (FAO) reports including the State of the World's Biodiversity for Food and Aquaculture and the State of the World's Aquatic Genetic Resources for Food and Aquaculture [39,40]. Genetic biodiversity therefore plays an fundamental role in a wide range of global sustainable development goals [41].

Genetic Diversity in the Decade of Ocean Science Implementation Plan

The Decade of Ocean Science Implementation Plan includes an overview of the major threats and impacts to the world's oceans. Yet the document does not explicitly highlight the significance of and threats to marine genetic diversity. The importance of the maintenance of biodiversity is acknowledged, however, failing to differentiate between genetic and species diversity has commonly led to genetic diversity being overlooked in policy and governance [38,42]. We believe that the Implementation Plan should explicitly include the maintenance of both genetic diversity and species diversity in its scope for biodiversity. Specifically, Paragraph 2, which describes the current status and threats to the world's oceans, should explicitly acknowledge the loss of genetic diversity and the threat that this poses to the maintenance of biodiversity. For example, including:

> "…continued warming on this scale is projected to lead to large-scale disappearance of corals and other highly productive ecosystems that are a cornerstone of the world's biodiversity, and a source of food and livelihoods for hundreds of millions of people. ***The erosion of both***

> ***genetic and species diversity through human impacts may further compromise the resilience and evolutionary capacity of the world's ocean ecosystems*** [16]. Deoxygenation resulting from the combined effects of increased nutrient loads and ocean warming is creating 'dead zones' and low oxygen zones throughout the ocean."

Secondly, we feel strongly that "the ocean we want" should include genetically diverse and resilient populations, as well as a rich diversity of species and ecosystems. This should be reflected in the Decade Outcomes, in particular Outcome 2 which relates to the health of the ocean's ecosystems, but also in Outcome 3 which aims for "A productive ocean supporting sustainable food supply and a sustainable ocean economy", both of which are intrinsically supported by genetic diversity. To this aim we suggest that the following amendments be made to Outcomes 2 and 3:

> "**Outcome 2: A healthy and resilient ocean where marine ecosystems are understood, protected, restored and managed.** Degradation of marine ecosystems is accelerating due to unsustainable activities on land and in the ocean. To sustainably manage, protect or restore marine and coastal ecosystems, priority knowledge gaps of ecosystems, and their reactions to multiple stressors, need to be filled. ***The maintenance of genetic and species biodiversity should also be considered a central component in maintaining resilience and adaptive capacity in marine ecosystems*.** This is particularly true where multiple human stressors interact with climate change, including acidification and temperature increase. Such knowledge is important to develop tools to implement management frameworks that build resilience, recognise thresholds and avoid ecological tipping points, and thus ensure ecosystem functioning and continued delivery of ecosystem services for the health and wellbeing of society and the planet as a whole."

> "**Outcome 3: A productive ocean supporting sustainable food supply and a sustainable ocean economy.** The ocean is the foundation for future global economic development and human health and wellbeing, including food security and secure livelihoods for hundreds of millions of the world's poorest people. Knowledge and tools to support the recovery of wild fish stocks, deploy sustainable fisheries management practices, and support the sustainable expansion of aquaculture, ***while protecting and maintaining essential genetic and interspecific biodiversity within ecosystems***, will be essential. The ocean also provides essential goods and services to a wide range of established and emerging industries including extractive industries, energy, tourism, transport and pharmaceutical industries. Each of these sectors has specific, priority needs in terms of increased knowledge, and support to innovation, technological development and decision support tools to minimise risk, avoid lasting harm, and optimise their contribution to the development of a sustainable ocean economy. Governments also require information and tools, for example via national accounts that incorporate ocean indicators, to guide development of sustainable ocean economies and promote marine sectors. ***Proposed indicators at all levels of biodiversity, including genetic diversity, should also be implemented***."

Finally, these aims should be incorporated into the Action Framework and relevant Ocean Decade Challenges to ensure that these are translated into meaningful actions. Differentiating between genetic and species biodiversity in Ocean Decade Challenge 2 in particular would ensure that genetic diversity over the next decade would not be overlooked in the sustainable development, management and conservation of our oceans:

> **"Challenge 2:** Understand the effects of multiple stressors on ocean ecosystems, and develop solutions to monitor, protect, manage and restore ecosystems and their ***genetic and species*** biodiversity under changing environmental, social and climate conditions."

Conclusions

The importance of genetic diversity in maintaining ecosystem function and evolutionary potential cannot be overstated. By explicitly recognising and including genetic diversity in its target Outcomes and Challenges, the Decade of Ocean Science framework can ensure that genetic diversity is considered in biodiversity assessments in the next ten years of ocean research, development and management. The visibility and reach of the Decade Implementation plan, to governmental, legislative, NGO, and stakeholder groups, will help raise awareness and increase familiarity with the significance of genetic diversity for biodiversity maintenance and sustainable development. The acknowledgement of the importance of genetic diversity in the Implementation Plan can act as the platform from which further actions can be developed and implemented, be they research, management and conservation plans, or further outreach and dissemination. Ultimately, the inclusion of genetic diversity in the Decade of Ocean Science's Challenges and Outcomes will facilitate and support the maintenance of genetic biodiversity in the coming decade. In doing so, the Decade of Ocean Science will further achieve its core goal of supporting ecosystem resilience and adaptability in the face of climate change alongside the sustainable development of the ocean for our continuing needs.

Citations

1. IOC. Revised Draft Implementation plan for the United Nations Decade of Ocean Science for Sustainable Development. (2020).
2. Hoffmann, A. A., Sgrò, C. M. & Kristensen, T. N. Revisiting Adaptive Potential , Population Size , and Conservation. *Trends Ecol. Evol.* 32, 506–517 (2017).
3. Raffard, A., Santoul, F., Cucherousset, J. & Blanchet, S. The community and ecosystem consequences of intraspecific diversity: a meta-analysis. *Biol. Rev.* 94, 648661 (2018).
4. Randall Hughes, A., Brian, D., Johnson, M. T. J., Underwood, N. & Vellend, M. Ecological consequences of genetic diversity. *Ecol. Lett*. 11, 609–623 (2008).
5. Prieto, I. et al. Complementary effects of species and genetic diversity on productivity and stability of sown grasslands. *Nat. Plants* 1, 1–5 (2015).
6. Wood, C. M., McKinney, S. T. & Loftin, C. S. Intraspecific functional diversity of common species enhances community stability. *Ecol. Evol*. 7, 1553–1560 (2017).
7. Blanchet, S. et al. A river runs through it : The causes , consequences , and management of intraspecific diversity in river networks. *Evol. Appl.* 13, 1195–1213 (2020).
8. Koricheva, J. & Hayes, D. The relative importance of plant intraspecific diversity in structuring arthropod communities: A meta-analysis. *Funct. Ecol*. 1–14 (2018). doi:10.1111/1365-2435.13062
9. Jump, A. S., Marchant, R. & Peñuelas, J. Environmental change and the option value of genetic diversity. *Trends Plant Sci*. 14, 51–58 (2009).
10. Schindler, D. E., Armstrong, J. B. & Reed, T. E. The portfolio concept in ecology and evolution. *Front. Ecol. Environ.* 13, 257–263 (2015).
11. Hermisson, J. & Penning, P. S. Soft sweeps and beyond : understanding the patterns and probabilities of selection footprints under rapid adaptation. *Methods Ecol. Evol.* 8, 700–716 (2017).
12. Nosil, P., Soria-Carrasco, V., Feder, J. L., Flaxman, S. M. & Gompert, Z. Local and system-wide adaptation is influenced by population connectivity. *Conserv. Genet.* 20, 45–57 (2019).
13. Hoffmann, A. A. & Sgrò, C. M. Climate change and evolutionary adaptation. *Nature* 470, 479–485 (2011).

14. Martin, T. G. & Watson, J. E. M. Intact ecosystems provide best defence against climate change. *Nat. Clim. Chang.* 6, 122–124 (2016).

15. Theodoridis, S., Patsiou, T. S., Randin, C. & Conti, E. Forecasting range shifts of a cold-adapted species under climate change: are genomic and ecological diversity within species crucial for future resilience? *Ecography*, 41, 1357–1369 (2018).

16. Leigh, D. M., Friesen, V. L., Hendry, A. P. & Domínguez, E. V. Estimated six per cent loss of genetic variation in wild populations since the industrial revolution. *Evol. Appl.* 12, 1505–1512 (2019).

17. Miraldo, A. et al. An Anthropocene map of genetic diversity. *Science,* 353, 1532–1535 (2016).

18. Mimura, M. et al. Understanding and monitoring the consequences of human impacts on intraspecific variation. *Evol. Appl.* 10, 121–139 (2017).

19. Allendorf, F. W., England, P. R., Luikart, G., Ritchie, P. A. & Ryman, N. Genetic effects of harvest on wild animal populations. *Trends Ecol. Evol.* 23, 327–337 (2008).

20. Gurgel, F. C. D., Camacho, O., Minne, A. J. P., Wernberg, T. & Coleman, M. A. Marine Heatwave Drives Cryptic Loss of Genetic Diversity in Underwater Forests Article Marine Heatwave Drives Cryptic Loss of Genetic Diversity in Underwater Forests. *Curr. Biol.* 30, 1–8 (2020).

21. Berger-Tal, O. & Saltz, D. Invisible barriers : anthropogenic impacts on inter- and intra-specific interactions as drivers of landscape-independent fragmentation. *Philos. Trans. R. Soc. B* 374, 20180049 (2019).

22. Frankham, R., Bradshaw, C. J. A. & Brook, B. W. Genetics in conservation management: Revised recommendations for the 50/500 rules, Red List criteria and population viability analyses. *Biol. Conserv.* 170, 56–63 (2014).

23. Pinsky, M. L. & Palumbi, S. R. Meta-analysis reveals lower genetic diversity in overfished populations. *Mol. Ecol.* 23, 29–39 (2014).

24. Heino, M., Pauli, B. D. & Dieckmann, U. Fisheries-induced evolution. *Annu. Rev. Ecol. Evol. Syst.* 46, 461–480 (2015).

25. de los Santos, C. B. et al. Recent trend reversal for declining European seagrass meadows. *Nat. Commun.* 10, 1–8 (2019).

26. Bryan-Brown, D. N. et al. Global trends in mangrove forest fragmentation. *Sci. Rep.* 10, 7117 (2020).

27. Glover, K. A. et al. Half a century of genetic interaction between farmed and wild Atlantic salmon: Status of knowledge and unanswered questions. *Fish Fish.* 1–38 (2017). doi:10.1111/faf.12214

28. Olden, J. D., Poff, N. L., Douglas, M. R., Douglas, M. E. & Fausch, K. D. Ecological and evolutionary consequences of biotic homogenization. *Trends Ecol. Evol.* 19, 18–24 (2004).

29. Laikre, L., Schwartz, M. K., Waples, R. S. & Ryman, N. Compromising genetic diversity in the wild: Unmonitored large-scale release of plants and animals. *Trends Ecol. Evol.* 25, 520–529 (2010).

30. Teagle, H. & Smale, D. A. Climate-driven substitution of habitat-forming species leads to reduced biodiversity within a temperate marine community. *Divers. Distrib*. 24, 1367–1380 (2018).

31. Buonomo, R. et al. Predicted extinction of unique genetic diversity in marine forests of *Cystoseira spp. Mar. Environ. Res.* 138, 119–128 (2018).

32. Wernberg, T. et al. Genetic diversity and kelp forest vulnerability to climatic stress. *Sci. Rep*. 8, 1851 (2018).

33. Oliver, E. C. J. et al. Projected Marine Heatwaves in the 21st Century and the Potential for Ecological Impact. *Front. Mar. Sci.* 6, 1–12 (2019).

34. Arrieta, J. M., Arnaud-Haond, S. & Duarte, C. M. What lies underneath: Conserving the oceans' genetic resources. *PNAS* 107, 18318–18324 (2010).

35. Goecke, F., Klemetsdal, G. & Ergon, Å. Cultivar Development of Kelps for Commercial Cultivation - Past Lessons and Future Prospects. *Front. Mar. Sci.* 8, 1–17 (2020).

36. Bernatchez, L. et al. Harnessing the Power of Genomics to Secure the Future of Seafood. *Trends Ecol. Evol.* 32, 665–680 (2017).

37. CBD. Zero Draft of the Post-2020 Global Biodiversity Framework. *Conv. Biol. Divers.* 1–14 (2020).

38. Hoban, S. et al. Genetic diversity targets and indicators in the CBD post-2020 Global Biodiversity Framework must be improved. *Biol. Conserv.* 248, 108654 (2020).

39. FAO. The State of the World's Biodiversity for Food and Agriculture. *Comm. Genet. Resour. Food Agric.* (2019).

40. FAO. The State of the World's Aquatic Genetic Resources For Food and Agriculture. *Comm. Genet. Resour. Food Agric.* (2019).

41. Blicharska, M. et al. Biodiversity's contributions to sustainable development. *Nat. Sustain.* 2, 1083–1093 (2019).

42. Laikre, L. Genetic diversity is overlooked in international conservation policy implementation. *Conserv. Genet.* 11, 349–354 (2010).

Signatories (in alphabetical order)

- Fred W. Allendorf, University of Montana, USA
- Carl André, Tjärnö Marine Laboratory, Göteborg University, Sweden
- Frederick Archer, Scripps Institution of Oceanography, University of California San Diego, USA
- Louis Bernatchez - IBIS (Institut de Biologie Intégrative et des Systèmes), Université Laval, Canada
- Michael Burrows, Scottish Association for Marine Science, UK
- Melinda Coleman, Department of Primary Industries and National Marine Science Centre, Australia
- Pierre De Wit, Gothenburg University, Sweden
- Gonzalo Gajardo, Universidad de Los Lagos, Chile
- Will Goodall-Copestake, Scottish Association for Marine Science, UK
- Michael M. Hansen, Department of Biology, Aarhus University, Denmark
- Phil Hedrick, Arizona State University, USA
- Sean Hoban, The Morton Arboretum, USA
- Margaret Hunter, U.S. Geological Survey, USA
- Jennifer Jackson, British Antarctic Survey, UK
- Torild Johansen, Institute of Marine Research, Norway
- Kerstin Johannesson, Tjärnö Marine Laboratory, Göteborg University, Sweden

- Linda Laikre, Department of Zoology, Stockholm University, Sweden
- Adam Miller, Deakin University, Australia
- Einar Eg Nielsen, National Institute of Aquatic Resources, Technical University of Denmark
- David O'Brien, Scottish Natural Heritage, UK
- Rob Ogden, University of Edinburgh, UK
- Jennifer Ovenden, University of Queensland, Australia.
- Sílvia Pérez-Espona, The University of Edinburgh, UK
- Craig Primmer, Organismal and Evolutionary Biology Research Program, University of Helsinki, Finland
- Jonathan Puritz, University of Rhode Island, USA
- Nils Ryman, Department of Zoology, Stockholm University, Stockholm, Sweden
- Atal Saha, Department of Zoology, Stockholm University, Sweden
- Jonathan Sandoval-Castillo, Flinders University, Australia
- Gernot Segelbacher, University Freiburg, Germany.
- Ester Serrao, Centre of Marine Sciences of the University of Algarve, Portugal
- Kjersti Sjøtun, University of Bergen, Norway
- Dan Smale, Marine Biological Association of the United Kingdom, UK
- Michele Stanley, Scottish Association for Marine Science, UK
- Alex Innes Thomson, Scottish Association for Marine Science, UK
- Cristiano Vernesi, Fondazione Edmund Mach, Italy
- Eric Verspoor, University of the Highlands and Islands, UK
